# The JMJD Family Histone Demethylases in Crosstalk Between Inflammation and Cancer

**DOI:** 10.3389/fimmu.2022.881396

**Published:** 2022-04-26

**Authors:** Jia Yang, Yuan Hu, Binjing Zhang, Xiao Liang, Xin Li

**Affiliations:** ^1^ Department of Gynecology and Obstetrics and Pediatric Nephrology Nursing, Development and Related Disease of Women and Children Key Laboratory of Sichuan Province, Key Laboratory of Birth Defects and Related Diseases of Women and Children, Ministry of Education, West China Second Hospital, Sichuan University, Chengdu, China; ^2^ State Key Laboratory of Oral Disease, National Clinical Research Center for Oral Diseases, West China Hospital of Stomatology, Sichuan University, Chengdu, China

**Keywords:** cancer, inflammation, cancer therapeutics, JMJD family, histone demethylases

## Abstract

Inflammation has emerged as a key player in regulating cancer initiation, progression, and therapeutics, acting as a double edged sword either facilitating cancer progression and therapeutic resistance or inducing anti-tumor immune responses. Accumulating evidence has linked the epigenetic modifications of histones to inflammation and cancer, and histone modifications-based strategies have shown promising therapeutic potentials against cancer. The jumonji C domain-containing (JMJD) family histone demethylases have exhibited multiple regulator functions in inflammatory processes and cancer development, and a number of therapeutic strategies targeting JMJD histone demethylases to modulate inflammatory cells and their products have been successfully evaluated in clinical or preclinical tumor models. This review summarizes current understanding of the functional roles and mechanisms of JMJD histone demethylases in crosstalk between inflammation and cancer, and highlights recent clinical and preclinical progress on harnessing the JMJD histone demethylases to regulate cancer-related inflammation for future cancer therapeutics.

## Introduction

Inflammatory cells and inflammatory factors are important components of the tumor microenvironment (1–3). In solid tumors, hypoxia and acidic stress in tumor microenvironmental niches induces apoptosis of the fast-growing tumor cells, releasing cell fragments and chemokines and leading to inflammatory cell infiltration and secretion of inflammatory factors. Furthermore, extrinsic factors such as systematic diseases, infections, and smoking are also able to evoke and enhance tumor-associated inflammatory response processes. Accumulating evidence has suggested that both tumor-extrinsic and -intrinsic inflammation are closely linked to tumorigenesis, development, and therapy resistance (4–7). Recently, epigenetics, particularly the Jumonji C domain-containing (JMJD) protein family-mediated histone methylation mechanisms, have been identified as one of the most important mechanisms for regulating the occurrence and progression of malignant tumors. In addition, a number of JMJD proteins-targeted therapeutic strategies aiming to modulate inflammatory cells and their products have been developed in clinical or preclinical tumor models. In this review, we summarize the roles and mechanisms of JMJD protein family in cancer-related inflammation, and discuss the anti-cancer therapeutic approaches by modulating JMJD proteins-mediated inflammation.

## JMJD Histone Demethylases in Cancer-Related Inflammation

### Crosstalk Between Cancer and Inflammation

In 1800, Galenus was the first to hypothesize a correlation between inflammation and tumors, suggesting that tumors can arise in inflamed and damaged tissues (8). In the 19th century, researchers found that tumors usually occurred in chronic inflammation sites, and there were inflammatory cells in tumor biopsy tissues (9). This is the first human evidence supporting the relationship between cancer and inflammation, and these findings have been confirmed by epidemiological research where researchers found that individuals with chronic inflammation were more likely to develop cancer.

Indeed, it is estimated that 15-20% of cancers worldwide are associated with underlying infection and inflammation. Tumor-related inflammation can be divided into the following categories according to the inducement, mechanism, result and intensity: inflammation caused by microbial infection, inflammation caused by environmental exposure, inflammation secondary to tumor, and inflammation secondary to tumor treatment. Importantly, tumor-related chronic inflammation has been shown to be an important trigger factor that increases the risks of cancer and promotes the cancer progression (4). For example, inflammation caused by microbial infection, including persistent helicobacter pylori infection, is associated with gastric cancer and gastric mucosa-associated lymphoid tissue lymphoma. In addition, Hepatitis B or C virus infection increases the risk of hepatocellular carcinoma, and schistosome or bacillus infection has been associated with bladder cancer and colon cancer, respectively (10). Furthermore, inflammation caused by environmental exposure, including particulate irritants such as grass smoke, can lead to chronic obstructive pulmonary disease which has a high risk for lung cancer (11). Inhalation of asbestos or silica particles can also cause lung cancer, and the tumorigenicity of these particles is attributed to the inflammatory effect induced by inflammasome-produced interleukin (IL)-1β cytokine (12). Not surprisingly, administration of non-steroidal anti-inflammatory drugs has been reported to significantly reduce the morbidity and mortality of multiple types of cancer (13–15).

In addition to its role in cancer initiation, the inflammatory responses were also shown to play an important role in cancer progression and metastasis. It is well-established that except for the cancer cells themselves, the stroma, blood vessels, and infiltrating inflammatory cells in tumor microenvironment are also involved in the regulation of inflammation and cancer. Tumor-related inflammatory molecules are known to mainly include transcription factors (such as NF-κB, as well as the signal transduction activation transcription factor STAT3 and HIF-1α), chemotactic factors, cytokines (such as IL-1β, IL-6, IL-23 and TNF-α), cyclooxygenase, and inducible nitric oxide synthase (iNOS), etc. (16). Studies have shown that NF-κB and STAT3 transcription factors are the main regulators for promoting the production of pro-inflammatory cytokines as well as the persistence of important mediators of chronic inflammation, and thus involved in the occurrence and development of tumors (17–20). Furthermore, interactions between inflammatory cells, cancer cells and stromal cells within the tumor microenvironment exist through mutual induction, receptor regulation and biological effects (21, 22). In addition, there are a large number of active substances which act through their own functional properties or mediated signaling pathways to participate in the cellular biological events necessary for tumorigenesis and cancer development. Certain cytokines are known to activate a variety of signaling molecules controlling a wide range of cellular processes, and thus linking the inflammatory microenvironment with biological behaviors of tumor cells to precede cancer development. For example, IL-6, IL-8, IL-10, IL-18, TGF-β and other cytokines act as indispensable factors to promote inflammatory responses for cancer progression (23). Studies have also shown that IL-6 is known to have a direct effect on both tumor cells and tumor inflammatory microenvironment, and is associated with several tumor types, including lymphoma, mesothelioma, lung, breast, pancreatic, colon, prostate, and stomach cancer (24–27). It has been demonstrated that epigenetic machineries are involved in regulating the secretion of inflammatory cytokines or the activation of signaling pathways. Taking transforming growth factor TGF-β as an example, the binding of TGF-β and corresponding cell membrane surface receptor triggers the transactivation of Smad protein, mediating upregulated or downregulated expression of the downstream genes involved in cancer promotion or inhibition (28, 29). Abnormal DNA methylation of tumor cells has also been found in a variety of tumor tissues, which can down-regulate or inhibit TGF-β-mediated signaling pathway by modulating TGF-β and its receptors (28). These results highlight the important roles of pro-inflammatory cytokines in tumor inflammatory microenvironment and cancer progression.

Therefore, inflammation, especially chronic inflammation, plays an important role in cancer initiation and progression. In the process of tumor formation, inflammatory reactions promote tumor growth, produce a microenvironment conducive to tumor growth, induce gene mutations, and promote angiogenesis. In the later stage of tumor development, inflammatory mediators affect tumor cells promote tumor growth, angiogenesis, and metastasis by modulating biological behaviors of tumor cells.

### Histone Methylation and Demethylation in Cancer-Related Inflammation

Epigenetics refers to the stable and heritable changes in gene expression or cell phenotype caused by reversibly modifying nucleotide or chromosome without changing DNA sequences. The concept of epigenetics was first proposed by Waddington, a British scientist, in 1942. Epigenetics refers to the change of gene expression level and function without the change of DNA sequence, and produce heritable phenotype. Epigenetics can be divided into two categories according to their action mechanisms to regulate gene expression, including the DNA methylation, histone modification, chromatin remodeling act by selective transcription and expression regulation of genes, as well as the non-coding RNAs act by post-transcriptional regulation of genes (30).

Histones are highly conserved proteins composed of H1, H3, H2A, H2B and H4, which form nucleosomes together with DNA (30, 31). As one of the basic structural components of chromosomes, histone is not only an important part of the DNA that participates in the composition of genetic material, but also plays an indispensable role in the regulation of gene expression. Histone modifications mainly include the following categories: phosphorylation (serine and threonine residues), methylation (arginine and lysine residues), acetylation (lysine residues), ubiquitination, glycosylation, ADP-ribosylation (lysine residues), ubiquitination and proline isomerization, etc. (32, 33). Chromatin is divided into heterochromatin and euchromatin according to the interaction of different modification types of histone tail residues with specific proteins (33). Histone modifications have been found to not only reversibly promote or inhibit the transcription and expression levels of corresponding genes, but also participate in several cellular processes such as cell proliferation, differentiation, and functions (34). The modifications of histones at individual sites occur following the corresponding reactions mediated by specific enzymes, and the modification status is dynamically regulated by the corresponding enzymes in the reaction. Histone methylation and demethylation, mainly occur on lysine (K) and arginine (R) residues of the histones, are one of the most studied epigenetic patterns in the tumor field (35–38). Histone methylation is the methylation of lysine (k) or arginine (s) residues at the N-terminal of H3 and H4 histones. Its function is to form and maintain the structure of heterochromatin, genomic imprinting, DNA repair, inactivation and transcription of X chromatin (39, 40). Histone methylation is mainly catalyzed by histone methyltransferase (HMT). Histone methylation can be involved in the maintenance of chromatin structure and the silencing or activation of gene expression. Histone demethylase, LSD1 can be roughly divided into two families: LSD (Lysine-specific Demethylase) and JMJD (JmjC Domain-containing Family). LSD1 can specifically remove the mono-dimethylation of H3K4 and H3K9. JmjC family can remove the modification of lysine trimethylation. In general, methylation of the K9, K27, and K20 residues of histone H3 normally induces heterochromatin formation and silencing of related genes, while methylation of the K4 residue of histone H3, K36 and K79 in turn regulates gene expression (39). Abundant experimental evidence has demonstrated that abnormal histone methylation regulates the biological functions of tumor cells, interfere with the immune response of tumor cells, and even the occurrence and development of certain tumors (41, 42). Taking the polarization process of tumor-associated macrophages (TAMs) as an example, it is currently believed that the transformation of TAMs from M1 type to M2 type is one of the important markers of tumor malignancy and development (43). It was found that DNA methyltransferase 3B (DNMT3b) was highly expressed in M1-type polarized macrophages, while it was lowly expressed in M2-type polarized macrophages, suggesting that abnormal methylation is involved in the transformation process of TAMs from M1-type to M2-type (44). Besides, the aberrant modification of histone H3K27 was also involved in the signaling pathways responsible for M2 polarization (45). Furthermore, several biological characteristics of tumor microenvironment, such as hypoxia, low pH, high interstitial hydraulic pressure, production of cytokines and proteolytic enzymes, and persistence of inflammatory response, are also important mechanisms for modulating inflammation-related histone methylation machinery during the occurrence and development of tumors.

### JMJD Family Histone Demethylases

The JMJD protein family is composed of 33 human members. The defining element of this protein family is the Jumonji C (JmjC) domain with a length of approximately 170 amino acids, containing an iconic HX (D/E) domain (46, 47). In addition, the JmjC domain also contains 2-oxoglutarate (2OG)/α-ketoglutarate binding sites similar to the catalytic domains of other 2OG-dependent oxygenases (48). This leads to the proved for the first time that the catalytic activity of the JmjC domain exists for HIF1-α (hypoxia inducible factor 1 subunit alpha inhibitor), indicating that it can hydroxylate asparagine residues (49, 50). Based on the structure and function relationships, such structural features confer JMJD proteins the catalytic activity to hydroxylate methylated lysine residues and then demethylate them (51). The histone lysine demethylase activity has been proven to exist in some members of JMJD proteins by further experimental studies (52, 53), leading to certain JMJD family proteins being renamed as lysine demethylases (KDMs). Several members of the JMJD protein family, such as JMJD2B, JMJD2C, JMJD2D, JMJD3, JMJD5, JMJD6, and JMJD8, have been shown to be closely involved in the development and progression of chronic diseases. Proteins containing JMJD have relatively small molecular weights and perform different functions by hydroxylating proteins and RNA, and these proteins are mostly epigenetic regulators mediating demethylation of the histone proteins.

## Functional Mechanisms of JMJD Histone Demethylases in Cancer-Related Inflammation

### JMJD Histone Demethylases and Cancer-Related Inflammation

Methylation modifications have been demonstrated to regulate the expression and function of key signaling molecules released by inflammatory cells and play a key role in certain cancer types. As we all know, the target genes of HIF-1 α, a key factor involved in the serial effects of hypoxia on tumor cells, include certain members of the Jumonji structures (JMJD)-containing histone demethylases (54). Pollard et al. (55) has found that siRNA targeting HIF-1 α can significantly reduce the expression of JMJD2B and JMJD2C proteins, suggesting that hypoxia can induce the activation of histone demethylase and promote the process of histone demethylation. Studies have also shown that hypoxia can induce disturbance of other epigenetic mechanisms such as DNA methylation, histone modification (56) and miRNA expression profile change in tumor cells (57). Besides, tumor stromal cells, especially the immune cells infiltrated locally by tumor, are known to produce a large number of inflammatory cytokines in the tumor microenvironment, and thus making the tumor cells in a long-term inflammatory state (58). This persistent inflammatory response plays an important role in altering epigenetic modification status and disturbance during cancer development. Studies have found that stromal cells in the myeloma microenvironment could inhibit miRNA-15a/-16 expression in bone marrow cells by secreting high levels of the inflammatory factor IL-6, thus weakening the inhibitory effects on tumor cell proliferation and participating in the occurrence of drug resistance in myeloma. In turn, overexpression of inflammatory cytokines TNF-α and IL-6 can induce promoter methylation of signal transduction inhibitor 1 and down-regulate its expression, thus alleviating the inhibition of inflammatory response in tumors (59). In highly metastatic prostate cancer, inflammatory factor CXCL1 activates the NF-κB signaling pathway, deacetylates histone H3 and H4, and down-regulates the expression of extracellular matrix glycoprotein fibulin-D1 (59). Together, these studies suggest that inflammatory cytokines and chemokines could modulate epigenetic machineries to trigger remodeling of the tumor microenvironment, affecting cancer development.

JMJD histone demethylases are abnormally expressed in inflammatory conditions and are responsible for regulating the expression and secretion of inflammatory mediators. A number of studies have shown a significant link between inflammation and epigenome reprogramming brought about by the JMJD family, and the JMJD family has been shown to be critical in chronic inflammation and autoimmune diseases, including atherosclerosis (60). In addition, demethylation of H3K4me3 and H3K27 is necessary for M1 macrophages to initiate inflammatory cytokine production and M2 polarization (61). Considering their pro-inflammatory roles and the current evidence on inflammation-cancer transformation, we speculated that JMJD family histone demethylases might be a key node in the crosstalk between inflammation and cancer, and their related functional mechanisms and therapeutic potentials are discussed. Despite the large number of members of the JMJD family, we found that only a limited number of members are associated with inflammatory tumors and their mechanisms are relatively well studied. Next, we will focus on the association between JMJD2B, JMJD2C, JMJD2D, JMJD3 and JMJD5 with inflammatory tumors.

### JMJD2B and Cancer-Related Inflammation

JMJD2B, also known as KDM4B, is a JmjC domain-containing histone demethylase belonged to the JMJD2 protein family. JMJD2B mainly targets the trimethyl of lysine 9 of histone H3 (H3K9me3) to catalyze its demethylation reaction. Such demethylation activity of JMJD2B has been found to play an important role in several biological processes, such as heterochromatin formation, X chromosome inactivation, gene transcription regulation, and stem cell differentiation (39, 60–64). Recently, JMJD2B has been recognized as a strong epigenetic modulator of inflammation (65), and have attracted much attention in the malignant progression of multiple types of cancer, such as breast cancer, gastric cancer, colorectal cancer, bladder cancer, and lung cancer (66–72).

As one of the most common malignant tumors worldwide, gastric cancer-related mortality ranked second amongst causes of death in cancer patients (73, 74). In detail, although the overall incidence of gastric cancer has fallen in the United States, its incidence in Asian countries is still on the high side (75). Among them, China is a high incidence area of gastric cancer, with an incidence of 34.6/100,000 and a mortality rate of 30.2/100,000 (76). Due to the insufficient early detection and diagnosis of gastric cancer, most patients have already entered the advanced or metastatic stage by the time of diagnosis. Besides, the currently available treatment options for advanced gastric cancer are still limited, surgery and combined chemotherapy have displayed insufficient effects on therapeutic effects, and the five-year survival among the gastric cancer patients remained very low (77). The age-standardized 5-year relative survival rate of gastric cancer patients in China was only 35.1% (78). Helicobacter pylori (*H. pylori*), a type of microaerobic Gram-negative bacteria that colonizes gastric mucosal epithelial cells, has been shown to be a risk factor for chronic gastritis, gastrointestinal ulcer, and gastric cancer (79). It has been classified as a class I carcinogen by the international agency for research on cancer (IARC) (80). Recent studies have suggested that *H. pylori* affected the invasion and metastasis of the gastric cancer by upregulating the expression of epithelial-mesenchymal transition (EMT)-related factors Snail 1, Slug and vimentin, partly relying on gastrin and matrix metalloproteinase 7 (*MMP7*), and enhancing the expression of Heparin-binding EGF-like growth factor (HB-EGF), thus promoting the EMT process in gastric cancer (81). In addition, *H. pylori* activates multiple intracellular pathways in epithelial cells, such as the NF-κB, β-catenin pathway and PI3K/AKT pathway, which induces increased inflammatory cytokine production, altered apoptosis rate, and deregulated epithelial cell proliferation and differentiation, finally leading to the oncogenic transformation of early human gastric cancer (78, 82–88).

Histone modifications have been reported to be involved in immunosuppression, thereby providing a preferred microenvironment for infection with either bacteria or viruses and further development of human diseases, including cancer (89). As a risk factor associated with the occurrence and development of gastric cancer, *H. pylori* infection-induced epigenetic disturbances, especially the histone modification alterations, are closely involved in regulating the expression of genes crucial for tumorigenesis and cancer development (90, 91). As a histone demethylase, JMJD2B has been reported to possess oncogenic activities in multiple types of human cancers (66, 70). More specifically, JMJD2B plays an important in gastric cancer initiation and progression by promoting the proliferation, survival, invasion and metastasis of gastric cancer cells (92, 93).

**Figure 1:**
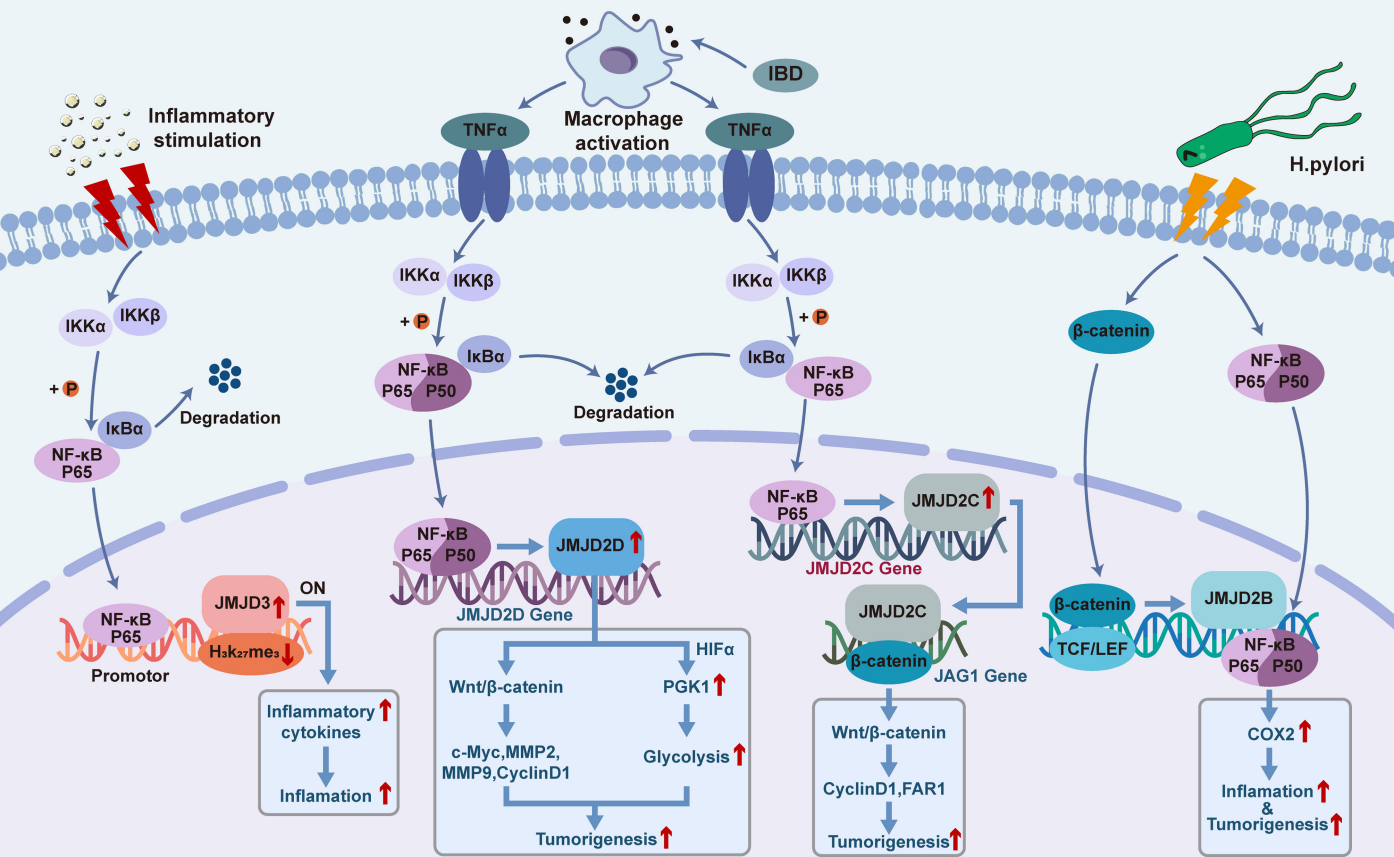
Functional roles and mechanisms of JMJD2B/C/D and JMJD3 in the crosstalk between inflammation and cancer.

It has been found that the mRNA and protein expression levels of JMJD2B in different gastric epithelial cells are significantly increased in response to *H. pylori* infection in a CagA-independent manner. Besides, it has been demonstrated that there is a potential binding site for β-catenin on the promoter region of *JMJD2B* (94). Indeed, amongst the multiple signaling pathways activated by *H. pylori*, β-catenin plays an important role in modulating dysregulated inflammation and gastric cancer progression (95). *H. pylori* infection induces the transcriptional activity of β-catenin, activates the nuclear LEF/TCF transcriptional factor, and regulates the expression levels of downstream target genes which are known to be involved in key cellular processes including cell cycle control, differentiation, and migration (96, 97). Meanwhile, in response to *H. pylori* infection, β-catenin has been shown to enhance the transcriptional activity of JMJD2B by recognizing the active region of the *JMJD2B* gene promoter (94). Besides, existing evidence has highlighted an interaction between JMJD2B and NF-κB in gastric cancer cells. It has been reported that *H. pylori* induces expression of *cyclooxygenase 2 (COX-2)* by activating the transcription factor NF-κB, which is a key regulator of immune and inflammatory responses, and thus further modulating the inflammatory response and contributing to the carcinogenesis of gastric cancer (98). Upon exposure to *H. pylori* infection, an increased binding of JMJD2B with NF-κB to *COX-2* promoter has been found to be coupled with the decrease of H3K9me3 and increase of H3K9me2 levels (94), and it can be concluded that JMJD2B could facilitate *H. pylori*-induced *COX-2* transcription. Indeed, multiple studies have demonstrated that *COX-2* is involved in *H. pylori*-induced inflammatory response and tumorigenesis (99, 100). Furthermore, an overexpressed expression pattern of *COX-2* has been detected in primary gastric cancer, and has been shown to be closely related to the occurrence and distal metastasis of gastric cancer (101–104). Hence, the β-catenin-JMJD2B-COX-2 signaling cascade described above is a crucial JMJD2B-dependent pathogenic mechanism responsible for the *H. pylori*-induced initiation and development of gastric cancer (**Figure 1**).

Besides, JMJD2B has also been associated with gastric cancer through other mechanisms. When exposed to hypoxia and radiation, histone H3 methylation of gastric adenocarcinoma cells was in a state of dynamic transformation, and JMJD2B-mediated modification mechanisms function to enhance the expression of *cyclin A1 (CCNA1)* and further facilitate the growth of gastric adenocarcinoma cells under hypoxia and radiation stress (67). These results suggested that histone modifications catalyzed by JMJD2B played an important role in the pathogenesis of gastric cancer under environmental stress, and this protein could become a potential target for the future development of treatment options for gastric cancer.

### JMJD2C and Cancer-Related Inflammation

*JMJD2C* gene is located in the p24.1 region of human chromosome 9. The total length of *JMJD2C* gene consists of 4239 bases, 13 different transcription products, including 22 exons and 3 promoters, forming a total of 6 variants. JMJD2C protein contains 1 JmjC, 1 JmjN, 2 phD-like zinc knuckle structures and 2 Tudor regions (105–107). In 2000, Y Ang et al. identified *JMJD2C* in the esophageal squamous cell carcinoma (ESCC) cells by comparative genomic hybridization, and named it as gene amplified in squamous cell carcinoma 1 (GASC1) (108). Subsequently, GASC1 was incorporated into the JMJD2 family by Katoh et al. (106), and is now also known as lysine specific demethylase 4C (KDM4C). JMJD2C can specifically remove lysine methyl groups from histone H3K9me3, H3K9me2, H3K36me3 and H3K36me2 through hydroxylation, thus participating in heterochromatin formation, gene imprinting, X chromosome inactivation, and gene transcription regulation (109). Up to now, studies on JMJD2C have attracted much attention on the following hot spots: its roles in controlling the stem cell fate (110–113), its involvement in the tumorigenesis and cancer development, as well as its potential for the development of novel anti-cancer drugs (105, 114, 115). Indeed, expression of JMJD2C has been found to be upregulated in breast cancer, gastric cancer, colorectal cancer, osteosarcoma, and other tumor types (116, 117).

Colorectal cancer, a common malignant tumor of the digestive tract system, ranks fifth in cancer incidence and mortality among China, and third in terms of incidence and mortality among all cancers in the United States (118). Interestingly, the incidence and mortality of males are significantly higher than that of females in China, while displaying basically the same in the United States (118, 119). Colorectal cancer develops slowly and is mostly transformed from chronic intestinal diseases. A large amount of evidence has indicated that colorectal cancer can be triggered by various factors, including gene mutation, epigenetic change, chronic inflammation, diet, and lifestyle (120). More specifically, multiple factors, including genetic factors (121, 122), family history (123), medical conditions such as chronic inflammatory bowel disease (124), type 2 diabetes (125), and previous cancer history of abdominal or pelvic radiation (126–129), have been reported to be potential risk factors of colorectal cancer in developing countries.

Currently, it is believed that colorectal cancer originates from colon cancer stem cells, and Notch and Wnt signaling pathways have been reported to be involved in the growth of the latter (130, 131). It has been shown that JMJD2C is involved in the interaction between Notch and Wnt pathways to promote the formation of stem cell-like spheres in colorectal cancer (132). In 2013, Yamamoto et al. evaluated the expression of *JMJD2C* gene in colorectal cancer cell line and found that *JMJD2C* was the downstream gene of β-catenin in Wnt pathway (132). Mechanistically, the expression level of JMJD2C protein was increased under the modulation of Wnt/β-catenin signaling pathway, and the protein product subsequently recruited β-catenin and co-located at the Jagged1 (*JAG1*) promoter for transactivation, thus enhancing the activation of Notch pathway and promoting the development of colorectal cancer (132). Kim et al. showed that *JMJD2C* gene was highly expressed in colorectal cancer and was necessary for the maximum growth of human colorectal carcinoma HCT-116 cell lines (133). It has also been reported that JMJD2C forms a complex with β-catenin and regulates the expression of several key downstream effectors of β-catenin signaling pathway. In addition, JMJD2C was found to upregulate the expression levels of *cyclinD1* and proto-oncogene *FAR1* to stimulate the proliferation of colon cancer cells (133). Besides, JMJD2C could positively regulate the anti-apoptotic factor BCL-2 to maintain the survival of cancer cells (133) (**Figure 1**).

### JMJD2D and Cancer-Related Inflammation

JMJD2D (also known as KDM4D), a protein containing 523 amino acids with a molecular weight of 60 kDa, also belongs to the JMJD2 subfamily of the JMJD family histone demethylases while its structure is different from that of JMJD2A-2C (134). In details, the JmjN domain is located at 17-59 amino acids of the N terminus and acts to regulate gene transcription, while the JmjC domain is located at 146-312 amino acids of the N terminus and functions to catalyze the demethylation reaction (105). This protein has been reported to play an important role in many biological processes. For example, it has been reported that interactions between JMJD2D and androgen receptor (AR) enhance the transcription activity of androgen-dependent genes and regulate the activation of androgen signaling (135). In addition, JMJD2D was found to be recruited to the DNA damage sites in a poly (ADP-ribose) polymerase 1 (PARP 1)-dependent manner, contributing to the DNA double-strand break repair process (136). JMJD2D could also promote the initiation complex formation and regulate DNA replication (137), it can function as an RNA binding protein to increase its chromatin-binding capability and thus enhance the H3K9me3 demethylation activity (138). Besides, JMJD2D has been shown to bind with p53 to enhance the transcriptional activity of P21 (139).

Studies have revealed that JMJD2D can promote the growth and metastasis of colorectal cancer by enhancing the Wnt pathway and glycolysis, while inhibiting JMJD2D can prevent the occurrence and development of colorectal cancer (140) (**Figure 1**). And, overexpression of JMJD2D could activate the Wnt/β-catenin signaling pathway, causing upregulation of the downstream target genes of Wnt signaling such as *c-myc*, *cyclin D1*, *MMP2*, and *MMP9* to promote the development of colorectal cancer (141). Under the condition of inflammatory bowel disease (IBD)-induced colon injury and intestinal bacterial translocation, activation of macrophages and secretion of inflammatory cytokine TNF-α trigger the activation of NF-κB signaling, leading to enhanced expression of JMJD2D in the colon epithelial cells. Meanwhile, JMJD2D has been shown to increase the transcriptional activity of phosphoglycerate Kinase 1 (PGK1) by interacting with HIF1-α, thus enhancing glycolysis to facilitate the progression of colorectal cancer. Further investigations on the functional role of JMJD2D histone demethylase activity in patients with colorectal cancer are imperative.

### JMJD3 and Cancer-Related Inflammation

JMJD3, also known as KDM6B, is another member of the JMJD family with H3K27me3 histone demethylase activity (142). The human *JMJD3* gene is located at 17p13.1 and encodes a protein consisting of 1682 amino acids with a molecular weight of 176632 Da. JMJD3 is located in both cytoplasm and nucleus, and the JmjC domain is indispensable for its H3K27me3 demethylation activity (143–145). It has been reported that this protein is involved in multiple cellular processes, including tumorigenesis and stem cell fate decision (143–146). Although JMJD3 is well-known for enzymatic activity to catalyze the conversion of H3K27me3 and H3K27me2 to H2K27me1, JMJD3 has been demonstrated to affect the proliferation of cancer cells and stem cells in either demethylation-dependent or -independent manner (147, 148). And, JMJD3 has been found to have both pro-cancer and anti-cancer properties by modulating the senescence and apoptosis of cancer cells (147).

JMJD3 plays an important role in the regulation of inflammation by affecting the polarization of macrophages, the differentiation of T cells, and the secretion of inflammatory factors. For example, it has been reported that serum amyloid A could upregulate JMJD3 *via* toll-like receptor (TLR) 2 and TLR4 pathways, thereby activating the secretion of proinflammatory cytokines of macrophages such as IL-1β, IL-8, and TNF-α (149). Besides, JMJD3 has been shown to mediate lipopolysaccharide-induced inflammation by directly targeting and promoting the transcription of important inflammatory factors such as *TNF-α, IL-6, IL-1β, COX-2*, and *ICAM-1* (150).

As mentioned above, NF-κB is a key coordinator of innate immunity and inflammation, and an important endogenous tumor promoter (19). It has been demonstrated that in tumor initiating cells, tumor cells, or inflammatory cells, NF-κB might function by acting as the downstream of microbial or tissue damage perception through the TLR signaling pathway as well as the signaling pathways mediated by the inflammatory cytokines TNF-α and IL-1β. In tumor cells, epithelial cells and inflammatory cells that are at risk of carcinogen transformation, NF-κB activates the prostaglandin synthesis pathway (such as *COX-2*), iNOS, and angiogenic factors. Besides, another important functional mechanism of NF-κB in tumor cells is to promote cell survival by modulating the expression levels of anti-apoptotic genes such as *BCL-2.* Furthermore, more and more studies have indicated the inter-connections and compensatory pathways between NF-κB and HIF1-α system (18, 151, 152), linking innate immunity with hypoxic response. And, NF-κB has been associated with the occurrence and development of tumors due to its regulatory effects on tumor-related inflammation in inflamed tissues (153, 154). Importantly, the inflammatory responses mediated by NF-κB have been closely associated with the pathogenesis of multiple tumor types. For example, NF-κB acts as a transcription factor to promote the expression of inflammatory cytokines IL-6 and IL-15, contributing to the development of colon cancer (155–158). NF-κB promotes the expression of inflammatory cytokines such as IL-17, IL-8 and TNF-α and facilitates the development of prostate cancer (159, 160). Moreover, NF-κB induces lymphatic carcinoma by promoting the expression of IL-6 and inflammatory COX-2, and activated NF-κB promotes the expression of inflammatory factors such as IL-22, TNF-α and iNOS to benefit the progression of hepatocellular carcinoma (161). Although many experimental and clinical studies have indicated that inflammation has tumor precursor activity, some evidence does not fit this general pattern. For example, obvious chronic inflammatory reactions such as psoriasis are not correlated with an increased risk of skin cancer (162). On the other hand, the presence of inflammatory cells is associated with a better prognosis in certain tumors or tumor subgroups as exemplified by eosinophils in colon tumors, and TAM in breast tumors and pancreatic tumor subgroups. These observations have suggested that inflammatory responses might also be tumor-suppressive (163). Indeed, it has been shown that properly activated macrophages, a typical component of cancer-related inflammation, can kill tumor cells and trigger a cancer-destructive inflammatory response surrounding the blood vessel wall, although their tumor-promoting properties are dominate in most cases (163). It is worth noting that there is evidence showing that NF-κB is important in determining the balance between tumorigenicity and anti-tumor properties of macrophages (164, 165), and modulating the function of NF-κB might be promising to improve the efficiency of cancer immunotherapy. Das et al. have reported that both NF-κB and JMJD3 were significantly upregulated in LPS-induced macrophage cell lines *in vitro* (166). With use of *JMJD3* gene knockout and stable transfection approaches, action mechanisms of the NF-κB-JMJD3 signal axis in monocytes were investigated; as a results, it was revealed that the deletion of *JMJD3* resulted in enrichment of H3K27me3 in promoter regions of NF-κB-related inflammatory target genes such as *monocyte chemoattractant protein-1 (MCP-1)* and *IL-1β*, and inhibited the transcription and expression of genes associated with inflammation (166). These results suggested that JMJD3 in the NF-κB-JMJD3 signaling axis could enhance the expression of NF-κB-related inflammatory genes by regulating H3K27me3 methylation levels. In mechanism, the activated NF-κB-JMJD3 signaling pathway recruits upregulated NF-κB/P65 and JMJD3 to H3K27ME3-rich transcription initiation regions, and the H3K27me3 conformation changes through the demethylation of Jmjd3, thus exposing the target gene transcription initiation binding site. The binding of the donor transcription factor-κB initiates the transcription process, resulting in the release of inflammatory mediators and adhesion molecules, contributing to the development of many chronic inflammation and inflammation-related cancers (**Figure 1**). Moreover, it has been reported that JMJD3 can not only regulate the expression of some inflammatory factors, but also bind to the promoters of some oncogenes or tumor suppressor genes, and play different roles in different stages of the disease development. For example, it has been well-known that functional loss of Ink4B/p15-ARF/p14-INK4A/p16 is an important inducing factor for tumorigenesis in multiple cancer types, and JMJD3 has been reported to promote the expression of tumor suppressors p16 and P14 (167). Studies have shown that JMJD3 is involved in inducing polarization of M2-like macrophages and thus participates in tumor progression (168). Taken together, considering the promoting roles of inflammatory factors in cancer (169), it might be reasonable to speculate that blocking JMJD3 may serve as an entry point for targeted therapy of chronic inflammation or even as a novel antitumor target for inflammation-related cancers.

### JMJD5 and Cancer-Related Inflammation

JMJD5 has two subtypes in human body, namely, subtype 1 and subtype 2 which containing 454 and 416 amino acid residues, respectively (170). Although JMJD5 (also known as KDM8) is a member of the JMJD family histone demethylases, this protein has been shown to function not only as a demethylase but also as a hydroxylase, playing an important role in cell cycle regulation, embryonic development, osteoclast differentiation, and circadian rhythm regulation (170, 171).

**Figure 2:**
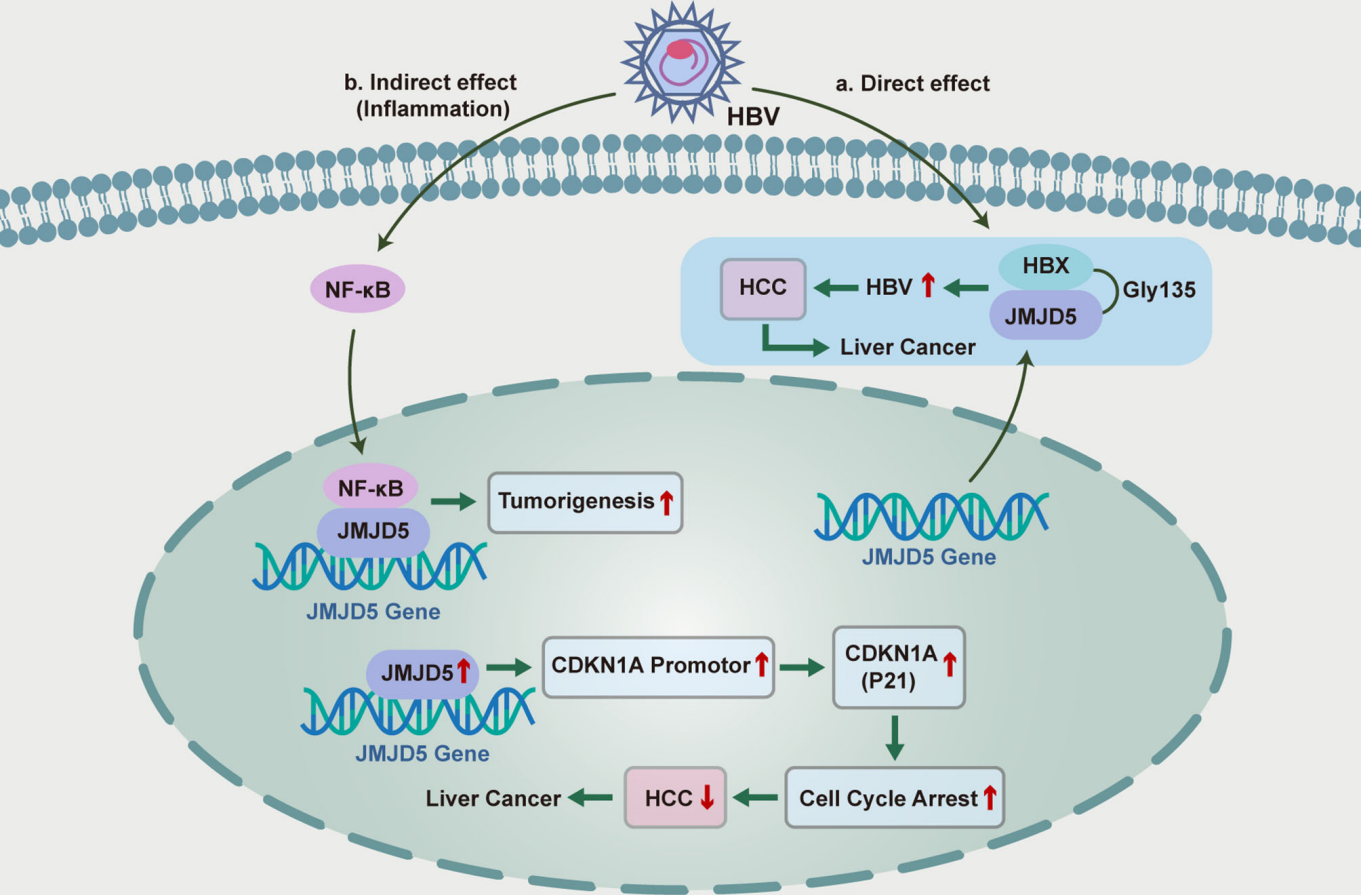
Functional roles and mechanisms of JMJD5 in the inflammatory regulation of HBC-induced hepatocellular carcinoma.

At present, although the pathogenesis of primary liver cancer is not clear, hepatitis B virus (HBV) infection and related immune response has been well-established to be involved in the pathogenesis of hepatocellular carcinoma (172). Kouwaki et al. has shown that JMJD5 could promote HBV replication through Gly135 interaction with HBV X protein (HBX), and thus JMJD5 is a novel HBX-binding protein responsible for the regulation of HBV replication (173). Although HBx has been confirmed to be localized in the cytoplasm and nucleus (174), JMJD5 is clearly localized in the nucleus, while co-expression of JMJD5 and HBX resulted in partial translocation of JMJD5 from the nucleus to the cytoplasm (173). To further confirm the role of JMJD5 and HBX interaction in HBV replication, researchers prepared JMJD5-knockout human “hemochromatotic” Huh7 cell lines and found that HBV replication was severely impaired in JMJD5 knockout cells (173). The interaction between JMJD5 and HBX was also found to be involved in post-transcriptional steps such as HBV capsid stabilization and aggregation. Gly135 of JMJD5 is a key amino acid residue that interacts with HBx, substitution of Gly135 by Glu could eliminate the repair effect of HBV replication by overexpressing JMJD5 in JMJD5-knockout cells (173). Moreover, expression of an enzyme deficient JMJD5 mutant that replaces His321 with Ala in the JmjC domain did not restore HBV replication in JMJD5 knockout cells (173). These results suggest that both the association of JMJD5 with HBX and the hydroxylase activity of JMJD5 are necessary for effective HBV replication. Besides, it has been suggested that JMJD5 could inhibit the proliferation and cell cycle progression of hepatocellular carcinoma cells by regulating P21 (175, 176), and thus affecting the development of hepatocellular carcinoma (**Figure 2**).

## Targeting the JMJD Histone Demethylases for Anti-Cancer Therapies

Herein, we summarized the preclinical or clinical progress of anti-cancer drug development by targeting the JMJD histone demethylases mentioned above.

### Pre-Clinical Trials Targeting the JMJD Histone Demethylases

With the gradual revelation of its role in tumorigenesis and development, JMJD3 has become a potential target in tumor therapy. At present, aiming at modulating the cancer promoting effect of JMJD3, specific small molecule inhibitors are synthesized to block the histone demethylation function of JMJD3 protein. For example, GSK-J4, a selective inhibitor of JMJD3 and UTX, has been proved to exert anticancer activity in a variety of tumor cell types, with its unique advantages of low action concentration, almost non-toxicity to normal cells, and reversible modification (177–179). As for colorectal cancer, Zhang J et al. found that GSK-J4 can strongly inhibit the characteristics of tumor initiation cells- and stem cells- related genes in colorectal cancer, reshape the epigenetic pattern, reduce the malignant phenotype of colorectal cancer cells, and sensitize them to chemotherapy (179). In the study of prostate cancer, Cao Z et al. found that GSK-J4 increased the H3K27me3 levels on *cyclinD1* promoter by inhibiting JMJD3 expression, and thus inducing cell cycle arrest and tumor cell death (180). These findings suggest that GSK-J4 could be able to reverse JMJD3-modulated biological behaviors of tumor cells. Although targeting JMJD3 has good antitumor effect, the role of JMJD3 and UTX in the same kind of tumor is not exactly the same (177), and the potential side effects of GSK-J4 should be taken into careful consideration. Moreover, JMJD3 has antitumor effect in some tumors, and enhancing the expression and activity of JMJD3 by vitamin D has been shown therapeutic effects in certain tumors such as colon cancer. However, there are currently few known JMJD3 activators, and, more efforts are needed to make the therapeutic effect more specific and safe.

JIB-04 is a small molecule compound showing inhibitory effects on the histone demethylation activity of JMJD proteins, without affecting other α-ketoglutarate dependent hydroxylase or histone modification enzymes (181). The therapeutic effects of JIB-04 have been evaluated by cell viability assays in a large cohort of cells, including human lung cancer cell lines, primary or immortalized non-tumorigenic human bronchial epithelial cells, as well as prostate cancer cell line, primary prostate stromal cells, and prostate epithelial cells. In addition, colony formation studies in multiple cell lines also confirmed that JIB-04 effectively inhibited tumorigenesis by interfering with anchoring independent cell growth. As for *in vivo* studies, Wang et al. constructed two xenograft mouse models of H358 and A549 in their experiment (181). In all drug-treated animals, tumor growth rates were significantly reduced in the JIB-04 drug group compared to the carrier group. Significant reductions in final tumor weight were also observed in both models, with no effect on overall body weight or overall health. Histological evaluation of major organs revealed no abnormalities except increased liver weight and liver vacuoles. Taken together, these findings suggested that this compound could alter the transcriptional programs responsible for the growth of cancer cells but not normal cells, inducing cancer-specific cell death. The mechanism and target therapies of JMJD protein family in inflammatory tumor were summarized in **Table 1**.

**Table 1:** The mechanism and target therapies of JMJD protein family in inflammatory tumor.

Gene	Inflammatory type	Cancer type	Mechanisms or pathways	Function	Targeted treatment for the JMJD family	Reference
JMJD2B	Helicobacter pylori	Gastric cancer	β-catenin pathway, NF-κB pathway	JMJD2B can be induced by H. pylori infection *via* β-catenin pathway. β-catenin directly binds to JMJD2B promoter and stimulates JMJD2B expression. Increased JMJD2B, together with NF-κB, binds to COX-2 promoter to enhance its transcription by demethylating H3K9me3 locally.	JMJD2B siRNA	(55, 94, 95),
JMJD2C	inflammatory bowel disease, IBD	Colorectal cancer	NF-κB pathway, Wnt/β-catenin pathway,JAG1, cyclinD1, Fra1	JMJD2C and β-catenin bind to the JAG1(Jagged 1) gene promoter to form a complex to promote colorectal cancer	JMJD2C siRNA, Caffeic acid, NOG2, JIB-04	(55, 132, 133, 181),
JMJD2D	inflammatory bowel disease, IBD	Colorectal cancer	NF-κB pathway, Wnt/β-catenin pathway,Glycolysis	Enhance Wnt pathway and glycolysis to promote colorectal cancer	JMJD2D siRNA, JIB-04,JMJD2D-knockdown	(136, 137, 139, 141, 181)
JMJD3	Colonitis	Colon Cancer	NF-κB pathway	Activating NF-κB pathway leads to chronic inflammation that leads to inflammation-related cancers	JMJD3 siRNA, GSK-J4	(149, 150) (177, 178),
JMJD5	Hepatitis B	Liver Cancer	Interaction of JMJD5 with HBx	JMJD5 as a novel HBx-binding protein regulating HBV replication	JMJD5 shRNA	(172)
	/	Liver Cancer	CDKN1A(P21)	JMJD5 regulates CDKN1A activity by binding to the CDKN1A promoter and thereby arresting the tumor cell cycle		(174)

### Clinical Trials Targeting the JMJD Histone Demethylases

So far, the only clinical trial of JMJD histone demethylases-based anti-cancer therapeutics is targeting JMJD2C. In view of the above, JMJD2C plays an important role in tumor development, and inhibition of this enzyme activity might hinder the proliferation and other biological behaviors of tumor cells (182). High expression of JMJD2C has been observed in a portion of clinical samples from patients with esophageal squamous cell carcinoma (ESCC), with its expression levels positively correlated with lymph nodes and tumor metastasis, as well as the low survival rate of ESCC (183). JMJD2C inhibitors available mainly include α-ketoglutaric acid analogues, such as N-oxalylglycine2 (NOG2), which can interfere with JmjC catalytic domain and affect JMJD demethylase activity through competitive antagonism against α-ketoglutaric acid (184, 185). Currently, a clinical trial evaluating caffeic acid as a JMJD2C modulator is being carried out in China, with the aim to investigate the efficacy and safety of caffeic acid in the treatment of advanced ESCC in China. Briefly, 240 patients with advanced ESCC will be randomly assigned to one of two groups: group A (caffeic acid treatment) or group B (placebo), followed for one year to determine the overall survival and progression-free survival of each group. Although the results of the clinical study have not yet been published, this trial might provide a paradigm for JMJD proteins-based precise targeted tumor therapy in the future. Considering the involvement of JMJD histone demethylases in cancer-related inflammation, targeting members of this protein family would contribute to the achievement of better outcomes for cancer therapeutics.

## Conclusions and Perspectives

Cancer-associated inflammation is one of the key features of cancer. Several members of the JMJD family histone demethylases have been shown to be involved in inflammation and its regulation and play an important role in many factors affecting tumor biology. From the perspective of tumor development, epigenetic regulation occurs at different stages of tumorigenesis, progression and metastasis, and is one of the important “tools” for the “dialogue” between tumor cells and inflammatory system. Inflammation can induce new epigenetic modifications in tumor cells, which in turn remodels the inflammatory tumor microenvironment. As an important part of epigenetics, abnormal patterns of the JMJD family-mediated histone methylation have been identified in the crosstalk between tumor progression and inflammatory processes, affecting the occurrence and development of different types of tumors. The complex relationship among inflammation, tumor and epigenetics also provides novel opportunities for the development of cancer therapeutics. Actually, targeted regulation of epigenetics for anti-cancer treatment has gradually attracted attention of researchers.

However, there are still problems to be solved when harnessing the histone demethylase activity of JMJD family proteins. First, histone demethylation activities have been shown to be partially responsible for the regulatory effects of JMJD histone demethylases on cancer-related inflammation, other involved mechanisms need to be further elucidated. Second, a large number of downstream targets of JMJD histone demethylases remain to be identified, which might make the therapeutic outcomes of targeted therapies unpredictable and induce treatment resistance. Third, there are only few specific small molecule compounds available to target the histone demethylation activity of JMJD proteins, and the crystal structure and structure-activity relationship of these proteins need to be clarified to facilitate high throughput drug screening in the future. The solution of the above problems could help us to have a deeper understanding on the crosstalk between inflammation and cancer, benefiting future development of personalized therapeutic strategies targeting the JMJD family histone demethylases.

## Author Contributions

Designed and wrote the manuscript: JY and YH. Revised the content: XiaL, XinL and BZ. Modified the language: XiaL and XinL. Draw the images: JY. All authors contributed to the article and approved the submitted version.

## Funding

This study was funded by grants from the National Natural Science Foundation of China (No. 81903033), the National Natural Science Foundation of China (No. 81902662), the National Natural Science Foundation of China (No. 81821002), Sichuan Science and Technology Program 2021YJ0011. This study was also supported by the Basic research project of Nursing Department of West China Second Hospital (HLBKJ202126) and Foundation of Development and Related Diseases of Women and Children Key Laboratory of Sichuan Province (Grant No.2022003).

## Conflict of Interest

The authors declare that the research was conducted in the absence of any commercial or financial relationships that could be construed as a potential conflict of interest.

## Publisher’s Note

All claims expressed in this article are solely those of the authors and do not necessarily represent those of their affiliated organizations, or those of the publisher, the editors and the reviewers. Any product that may be evaluated in this article, or claim that may be made by its manufacturer, is not guaranteed or endorsed by the publisher.
